# Size Distribution of Colistin Delivery by Different Type Nebulizers and Concentrations During Mechanical Ventilation

**DOI:** 10.3390/pharmaceutics11090459

**Published:** 2019-09-05

**Authors:** Ching-Yi Liu, Hsin-Kuo Ko, James B. Fink, Gwo-Hwa Wan, Chung-Chi Huang, Yu-Chun Chen, Hui-Ling Lin

**Affiliations:** 1 Graduate Institute of Clinical Medicine, College of Medicine, Chang Gung University, Taoyuan 33302, Taiwan; 2 Division of Respiratory therapy, Department of Chest Medicine, Taipei Veterans General Hospital, Taipei 11217, Taiwan; 3 School of Medicine, National Yang-Ming University, Taipei 11217, Taiwan; 4 Aerogen Pharma Corp., San Mateo 94402 CA, USA; 5 Department of Respiratory Care, Chang Gung University of Science and Technology, Chiayi 61301, Taiwan; 6 Department of Obstetrics and Gynaecology, Chang Gung Memorial Hospital-Taipei, Taipei 10507, Taiwan; 7 Department of Respiratory Therapy, College of Medicine, Chang Gung University, Taoyuan 33302, Taiwan; 8 Division of Thoracic Medicine, Chang Gung Memorial Hospital-Linko, Taoyuan 33301, Taiwan; 9 Institute of Emergency and Critical Care Medicine, National Yang-Ming University, Taipei 11217, Taiwan; 10 Department of Respiratory Therapy, Chiayi Chang Gung Memorial Hospital, Chiayi 61301, Taiwan

**Keywords:** inhaled colistin, jet nebulizer, vibrating mesh nebulizer, drug concentration, mechanical ventilation, inhaled drug mass, particle size distributions

## Abstract

Although aerosol delivery through mechanical ventilators has been used to administer various medications, little is known of administration with colistin. This in vitro evaluation aimed to evaluate size distribution of colistin delivery by different types of nebulizers and concentrations during mechanical ventilation. Colistin methanesulfonate (colistin) for injection was dissolved in 6 mL of distilled water to produce a low concentration (L; 156 mg) and a high concentration (H; 312 mg). A dose volume of 6 mL was placed in a vibrating mesh nebulizer (VMN) and a jet nebulizer (JN). The inhaled mass (mean ± SD) of the VMN-L (53.80 ± 14.79 mg) was greater than both the JN-L (19.82 ± 3.34 mg, *P* = 0.001) and JN-H (31.72 ± 4.48 mg, *P* = 0.017). The nebulization time of the VMN-L (42.35 ± 2.30 min) was two times longer than the JN-L (21.12 ± 0.8 min) or JN-H (21.65 ± 0.42 min; *P* < 0.001). The mass median aerodynamic distal to the endotracheal tube was within a similar range at 2.03 to 2.26 μm (*P* = 0.434), independent of neb or formulation concentration. In conclusion, the VMN-L yields greater inhaled mass than the JN with either concentration. Therefore, a standard nominal dose of colistin results in a higher delivered dose during mechanical ventilation with a VMN compared with a JN and may be considered the preferred device. If JN must be used, multiple doses of low concentration colistin may compensate for poor delivery performance.

## 1. Introduction

Colistin methanesulfonate (colistin) is one of the antibiotics used to treat gram-negative infection and hospital-acquired lower airway infection. It has also recently been used to treat gram-negative-induced ventilator-associated pneumonia (VAP) [1,2,3]. Colistin is primarily administered intravenously or through inhalation. However, evidence shows that systemically intravenous (IV) administration is only capable of low local concentrations in the lung, due to poor penetration [1,4]. Inhaled colistin is a proven method of efficiently targeting lung parenchyma with fewer systemic adverse effects [1,5]. Clinical studies of mechanical ventilation differ in reported outcomes, which may be due to differences in the amount of drug delivered to the lungs.

Our literature review found that colistin is administered either by IV, IV plus inhalation, or by inhalation alone, and that the effectiveness of colistin varies among patients [6]. While inhaled colistin has been associated with improved clinical cure rate and fewer ventilator days, other studies have reported no substantial benefits [6,7,8,9]. A large randomized control trial conducted by Haworth et al. revealed that the use of inhaled colistin to treat patients with bronchiectasis and chronic pseudomonas aeruginosa infection was safe and effective but had a low microbiological eradication rate [10]. 

While interest in using aerosolized colistin to treat intubated patients with VAP is increasing, little has been reported on the efficiency of inhaled colistin. Numerous factors influence aerosolized drug delivery through mechanical ventilation, including the ventilator settings, ventilator circuit, and choice of device, which generates different drug doses and particle sizes [11,12]. The dose of the drug delivered by the nebulizer is affected by the viscosity of the drug formulation, and antibiotic solutions tend to have greater viscosity [1,13]. Previous studies have indicated that jet nebulizers are capable of aerosolizing viscous solutions, such as dextran, and the higher viscosity of such drug solutions is associated with lower inhaled mass and smaller particle size [14,15]. However, the impact of heated gas with mechanical ventilation on aerosol particle size with different viscosities has not been reported.

A high dose of inhaled colistin has been proposed to treat VAP, and results have demonstrated its effectiveness with a low acute kidney injury rate [16,17,18]. However, the appropriate dosing and particle size distributions remain unclear, and guidelines for administering inhaled colistin during mechanical ventilation are yet to be established. The active ingredient of colistin is colistimethate sodium, which is typically manufactured in a dry powder form. When high doses of colistin are prescribed, clinicians may reduce the dilution volume to colistin powder and combine the higher concentration colistin solution in a nebulizer, so that treatment can be administered quicker. Different drug dilutions and concentrations may change the viscosity of the solution, which may alter the efficiency of delivery. This in vitro evaluation aimed to evaluate the size distribution of colistin delivery by different types of nebulizers and concentrations in an adult mechanical ventilation model.

## 2. Materials and Methods 

### 2.1. Lung Model

A Servo-i ventilator (Servo-I1, Getinge Group Co., Rastatt, Germany) was set in volume control with a tidal volume of 500 mL, a respiratory rate of 20 breaths/min, a positive end expiratory pressure of 5 cm H_2_O, and an inspiratory time of 1.0 s. Heated humidification (MR850, Fisher & Paykel Healthcare, Inc., Auckland, New Zealand) operating at 37 ± 1 °C was connected to a 7.5 mm endotracheal tube with an inline collecting filter or cascade impactor, and then connected to a lung model with compliance of 0.04 L/cm H_2_O and resistance of 5 cm H_2_O/L/s (Training & Test Lungs, Michigan Instrument Inc., Kentwood, MI, USA). The ambient temperature of the lab was 21 °C with 60–65% relative humidity. The endotracheal tube was wrapped with a heated pad to simulate surrounding tissue temperature and minimize reduction of gas temperature and associated condensate.

### 2.2. Study Design

To compare inhaled colistin delivery at different concentrations, colistin methanesulfonate (colistin) dry powder for intravenous injection (TTY Biopharm Co., Taipei, Taiwan), containing 2 million international units with 156 mg of colistin methanesulfonate in each vial, was used. Two concentrations were prepared for aerosol testing: low concentration (based on the standard label concentration for IV administration) with one vial (156 mg) in 6 mL of distilled water, and high concentration with two vials (312 mg) in 6 mL of distilled water, which is consistent with recommended fill volumes for optimal jet nebulizer (JN) delivery efficiency due to high residual drug in JNs [19].

**Figure 1:**
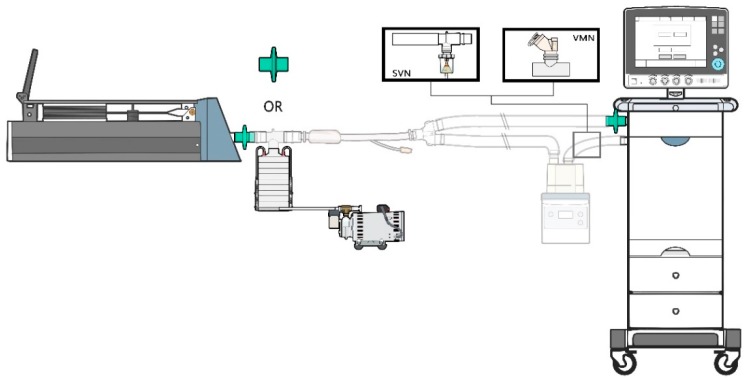
Experimental apparatus set-up. A ventilator was connected to a test lung via an endotracheal tube with an inline collecting filter or Andersen Cascade Impactor. SVN: small volume nebulizer, VMN: vibrating mesh nebulizer.

A vibrating mesh nebulizer (VMN, Aerogen Solo, Aerogen Ltd., Galway, Ireland) and a pneumatic jet nebulizer (reusable nebulizer NB-32400, Besmed Corp., Taipei, Taiwan) were placed at the inlet of a heated humidifier (Figure 1). The VMN was powered by its electronic controller (ProX, Aerogen Ltd., Galway, Ireland) and the JN was powered by oxygen at a flow of 8 L/min per manufacturer label. All experiments were performed using new nebulizers and were repeated five times (*n* = 5).

To determine the effect of increased concentration, the viscosity of the 2 dilutions was analyzed using a microviscometer (µVISC™, RheoSense Inc., San Ramon, CA, USA) with a chip measurement range of 0.2–100 mPa. The microviscometer was used with a temperature controller to maintain a temperature of 20 ± 0.5 °C.

### 2.3. Drug Delivery and Particle Size Distribution Measurement

The delivered dose, nebulization time, delivery efficiency, and particle size were measured as follows: 

#### 2.3.1. Delivered Drug Dose

A collecting filter (VADI Medical Technology Co., Taoyuan, Taiwan) was placed at the distal end of the endotracheal tube to collect the delivered drug. The filter was disassembled, eluted with 20 mL of distilled water, and then gently agitated for 2 min. A prior experiment identified this method of optimizing extraction of colistin at 92%. The delivered drug was analyzed using high-performance liquid chromatography (HPLC), and the percentage of the total dose was calculated. 

#### 2.3.2. Nebulization Time

The nebulization time was recorded from the beginning of nebulization until no aerosol could be observed for the VMN, and 30 s after the onset of sputter for JN. 

#### 2.3.3. Drug Delivery Efficiency

The drug delivery efficiency, defined as inhaled dose in mg and percentage divided by nebulization time, was used to compare the nebulizer efficiency in the 2 concentrations. 

#### 2.3.4. Particle Size Distribution 

Aerosols delivered distal to the endotracheal tube were sampled using an 8-stage Andersen Cascade Impactor (ACI, Thermo Fisher Scientific Inc., Waltham, MA, USA) for particle size distribution at ambient temperature of 20.5 °C with 56% relative humidity. The ACI was placed distal of the endotracheal tube, at the same position as the collecting filter. The ACI was operated at 28.3 L/min in the ventilator system, and an external gas was bled into the ventilator system to reach flow/pressure balance throughout inspiration and expiration. Prior to each test, the vacuum flow was validated using a flow meter (Mass Flow Meter 4040, TSI Incorporated, Shoreview, MN, USA). To prevent overloading of the collecting plates of the ACI, each sample was collected for 20 min. Drugs deposited on the collecting plates were eluted with 5 mL of distilled water and gently agitated for 2 min. The eluted drug from each stage was analyzed using HPLC. The mass median aerodynamic diameter (MMAD), geometric standard deviation (GSD), and fine particle fraction (%) defined as particle size ranging from 1.1 to 4.7 μm were calculated.

### 2.4. Drug Analysis

To minimize the hydrolysis process of colistin, the eluted drug samples were stored at 4 °C according to a previously described analytical method [20,21]. Colistin was analyzed using a validated HPLC method and an Agilent HP 1260 (Agilent Technologies, Santa Clara, CA, USA) equipped with an ultraviolet detector (G1312B), auto-sampler (G1329B), degassing unit (G1322A), and column oven (G1316A). The analysis was performed using an Agilent C18 column with a particle size of 5 μm (Agilent Eclipse XDB-C18). The column oven was set at 10 °C. A trifluoroacetic acid 0.05% (*v*/*v*) water solution (Sol A) and acetonitrile (Sol B) mixture were used as the mobile phase in the gradient elution. Colistin was analyzed at a detector wavelength of 210 nm. The injection volume was 20 mL, and a flow rate of 1.00 mL/min was used. The colistin retention time was 17 min. Solutions of colistin in water at 0.2, 0.4, 2, 4, 8, and 20 mg/mL were prepared using the United States Pharmacopeia (USP) colistin methanesulfonate reference standard. The colistin met the USP test specifications. The linearity ranged from 0.25 to 1.2 mg/mL (*R*^2^ = 0.9986) based on USP grade colistin (Sigma-Aldrich Corp., St. Louis, MO, USA). The percentage of colistin recovered from the working standard solution was determined at 94.5% with a confidence interval of 0.95.

### 2.5. Statistical Analysis

The delivered drug dose was considered in terms of mass and calculated as a percentage of the total dose. Data were analyzed using the Statistical Package for the Social Sciences version 23.0 (IBM Inc., Armonk, NY, USA) and expressed as means ± standard deviation. Comparisons of the drug dose and particle size were conducted using a one-way analysis of variance with a Scheffe post hoc test. The viscosity of the 2 concentrations was compared through analysis with independent *t*-tests. Linear regression was used to determine the correlation between the delivered drug dose and drug concentration. A P-value of 0.05 was considered statistically significant.

## 3. Results

### 3.1. Viscosity of Colistin Solutions

The viscosity of the low concentration solution was 1.234 ± 0.001 mPa. The viscosity of the high concentration was 1.310 ± 0.002 mPa (*P* < 0.001). A correlation was found between the inhaled drug dose and the viscosity of the two concentrations with JN (*R*^2^ = 0.715; *P* = 0.21).

### 3.2. Nebulizer Performance

The nebulization time of the VMN-H required 82 min; therefore, subsequent experiments with VMN were limited to a low concentration. Table 1 shows the inhaled mass (mg), inhaled dose as a percentage of the nominal dose, nebulization time, and delivery efficiency among the three groups. The inhaled mass of the VMN-L (53.80 ± 14.79 mg) was significantly greater than that of the JN-L (19.82 ± 3.34 mg, *P* = 0.001) and JN-H (31.72 ± 4.48 mg, *P* = 0.017). 

The nebulization time of the VMN-L (42.35 ± 2.30 min) was approximately two times longer than either JN-L (21.12 ± 0.86 min) or JN-H (21.65 ± 0.42 min; *P* < 0.001). In terms of delivery efficiency per min, the JN-H had the highest mg/min while JN-L had the lowest. By contrast, the VMN-L had the highest proportion of the dose delivered per minute, with three times greater delivery efficiency than JN-H.

### 3.3. Particle Size Distribution

**Figure 2:**
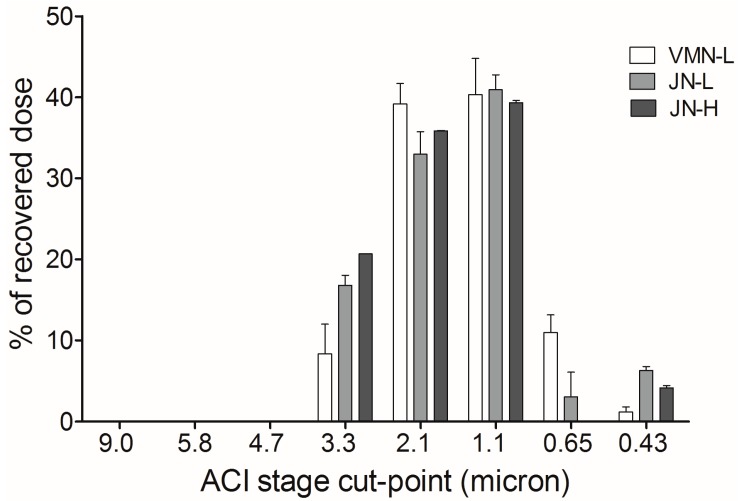
Particle size distribution at each stage of the Andersen Cascade Impactor.

Table 2 shows the particle size distributions of nebulized colistin exiting the endotracheal tube during mechanical ventilation, including the MMAD, GSD, fine particle percentage (%), and the total sampling dose. Drugs were only detected on Stages 3 to 7 (cut-point of <0.43 µm). The MMAD, GSD, and fine particle fraction were similar. Figure 2 illustrates drug deposition at each stage of the ACI sampling. 

## 4. Discussion

This in vitro study evaluated drug delivery of nebulized colistin during simulated adult mechanical ventilation with two nebulizer types and two concentrations of colistin. Our findings reveal that VMN-L produces a greater total of emitted and inhaled drug doses but requires double the nebulization time of JN for both concentrations. The particle size distribution at the end of the endotracheal tube during mechanical ventilation was similar regardless of the type of nebulizer and drug concentration.

### 4.1. Delivered Drug Dose

The drug dose deposition to the lower airway during mechanical ventilation is associated with the initial dose, inhaled mass, and particle size distribution [11]. Vibrating mesh nebulizers have demonstrated two to three times the delivered drug dose with a bronchodilator compared to JN [22]. Similarly, our results show that the inhaled mass of the VMN-L at 34.44% is two to three times greater than JN-L (12.69%) and JN-H (10.15%). 

In this study, due to the different nebulization times, we introduced a calculation of efficiency defined as the drug mass divided by the nebulization time as a comparator. With a double drug dose of initial charge, the JN-H yields the greatest delivery efficiency in mg/min. By contrast, the JN-H has a lower delivery efficiency %/min (0.60 ± 0.10) compared to the VMN-L (0.81 ± 0.20). The only significant difference is between the JN-H (0.47 ± 0.06 %/min) and the VMN-L (*P* = 0.015). Previous studies have reported that the %/min of albuterol delivered by a JN was 0.52 %/min, whereas the delivery by a VMN was 2.14 %/min [23,24]. The delivery efficiency of the JN was comparable to our data; however, the delivery efficiency of the VMN-L in our study with colistin is 0.81 ± 0.20 %/min lower than that with salbutamol, as found by previous studies. Increased drug viscosity has been known to influence the delivery time of both JN and VMN. Finlay et al. reported that the higher the concentration and viscosity of nebulized dextran by a JN, the lower the inhaled dose, while Zhang et al. found increasing viscosity significantly reduced nebulizer output [15,25]. Ghazanifari investigated the effect of fluid physiochemical properties on VMN and found that increased viscosity of fluid reduced the output rate, resulting in prolonged nebulization time [26]. 

Colistin, a family of polymyxins composed of amino compounds linked to a fatty acid, is likely to foam during nebulization; the longer the nebulization, the more foam is produced. Birkun et al. assessed the influence of a natural surfactant preparation of antibiotics in vitro and found that a colistin solution generated larger bubbles throughout the experiment [27]. The foaming effect appears to have resulted in a longer nebulization time. In summary, the VMN generates greater delivery efficiency but is slower with Colistin than reported in previous studies conducted with salbutamol.

### 4.2. Particle Size Distribution

Studies have characterized nebulized colistin with different strengths, at 33–75 mg in 4–6 mL of saline, reporting that MMAD varied between 1.4 and 3.2 μm emitted from the same nebulizer in spontaneous breathing models [28,29]. Others have identified that the MMAD of an aerosol exiting from an endotracheal tube during ventilation is limited to approximately 2 ± 0.5 μm, suggesting that the circuit and artificial airway act as a baffle, filtering out particles greater than 2 μm as they pass through the circuit and airway to the patient. This phenomenon can be demonstrated by narrow GSDs [29]. This explanation is consistent with our finding of reduced GSDs. As larger particles are removed from an aerosol, the GSD is reduced to a level approaching monodisperse aerosol (1.4 μm) [30].

Yang et al. measured Combivent delivered to the distal end of an endotracheal tube and found that the MMAD of the VMN was 1.57 µm and 1.9 µm for a JN [31]. The marginally larger MMAD found in our study with colistin may be explained by the different flow settings and ventilatory parameters used. Our study set the ventilator parameters, resulting in an inspiratory flow of 42 L/min vs. 60 L/min compared to Yang’s study. As the ventilator flow increased, greater impaction occurred in the circuit and airway, resulting in smaller particle sizes being emitted from the end of the endotracheal tube. Other than differences in the formulations of the two drugs, the placement of the nebulizer may have contributed to a slightly larger MMAD. Yang et al. placed the nebulizer 15 cm from the Y piece in the inspiratory limb, whereas we placed the nebulizer proximal to the heated humidifier chamber. When aerosols traveled the longer distance through the inspiratory limb, hygroscopic growth occurred [31,32,33].

To verify the ACI sampling model in our study, the total dose collected by the ACI was compared to the inhaled mass collected by the filter. The sampling time for the ACI was set at 20 min, so as not to overload the stages. By contrast, the nebulization time for the filter lasted until no aerosol could be observed for VMN and 30 s after the onset of sputter for JN. The collected dose from the ACI stages for the VMN was 18.86% of the total dose, approximately half of the inhaled mass (34.44%), which correlated to half of the collecting time (42.35 min). Similarly with the JN, the collected dose from the ACI was comparable to the inhaled mass, but the sampling time of the ACI was slightly shorter (JN-L: 12.07% for ACI vs. 12.69% for inhaled mass; JN-H: 10.55% for ACI vs. 10.15% for inhaled mass). Furthermore, during the vacuum pump sampling, the ventilator function was constantly monitored for inspiratory pressure and tidal volume, which remained unchanged throughout the experiments.

### 4.3. Clinical Implication

This study demonstrated the range of delivery efficiency achieved during mechanical ventilation with colistin via two commonly used types of nebulizers. The lung dose of colistin to optimize clinical response in mechanically ventilated adults has not been established. Device selection should be based on multiple factors including the amount of the dose delivered, cost of nebulizer, cost of drug, and time required to administer. The cost of medication is a key factor in aerosol device selection. In the US, the price of colistin is approximately $25/vial. To achieve a similar delivered dose of one vial with the VMN-L (53.8 mg), the JN-H would require more than the two vials studied. On the basis of two treatments per day, two to four additional vials ($50–$100) would be required with JN per day, increasing medication costs by $350–$700 for a 7-day course of colistin. In this scenario, the VMN which costs $45 and can be used for up to 28 days, would be the most cost-effective strategy. As for staff time, JN-L would require additional doses, and require the nebulizer to be removed from the circuit and washed or replaced after each dose, in contrast to the VMN, which can remain in the ventilator circuit for up to 28 days without cleaning. For ambulatory patients’ treatment, time can be critical for adherence; however, for patients intubated during mechanical ventilation, the additional dosing time with VMN is less of a factor than drug cost saving. Based on these factors, the clinical use of VMN-L is recommended.

### 4.4. Limitations

This study has certain limitations. VMN-H was not compared because of the long nebulization time. It should be kept in mind that our low concentration is actually the standard label concentration for IV administration, and the effects of aerosol administration with higher concentrations were not considered due to the potential impact on patient safety. Previous studies illustrate that nebulized aerosol delivery is influenced by the formulation of aqueous solutions [34,35]. Compared to saline, the physical properties of colistin include higher osmolality, lower surface tension, and higher viscosity. While our study only measured viscosity, further studies are desired on the influence of osmolality and surface tension on drug delivery distal to the endotracheal tube. Additionally, we measured the delivered colistin, which requires a conversion to colistin base activity for the bactericidal effect. The therapeutic effect of colistin, the delivered drug dose, and particle size warrant further evaluation.

## 5. Conclusions

The present lung model study shows that the VMN with a low concentration of colistin yields a greater inhaled mass of colistin delivered distal to the endotracheal tube than JN with either concentration. A standard single vial nominal dose of colistin results in a higher delivered dose during mechanical ventilation with a VMN compared to a JN, with a high concentration consisting of two vials. If JN must be used, multiple doses of colistin may compensate for poor delivery performance. The dose of colistin required for the therapeutic effect and bacterial eradication rate warrants further clinical evaluation.

## Figures and Tables

**Table 1:** Nebulizer performance on the delivery of colistin.

Variables	VMN-L	JN-L	JN-H	*P*-Value
Inhaled mass (mg)	53.80 ± 14.79	19.82 ± 3.34 *	31.72 ± 4.48 *	<0.001
Inhaled mass (%)	34.44 ± 9.47	12.69 ± 2.14 ^†^	10.15 ± 1.43 ^†^	<0.001
Nebulization time (min)	42.35 ± 2.30	21.12 ± 0.86 ^‡^	21.65 ± 0.42 ^‡^	<0.001
Delivery efficiency (mg/min)	1.27 ± 0.32	0.94 ± 0.17	1.46 ± 0.20 ^§^	0.023
Delivery efficiency (%/min)	0.81 ± 0.20	0.60 ± 0.1	0.47 ± 0.06 ^‖^	0.014

Note: Data are presented as means ± standard deviation; VMN: vibrating mesh nebulizer; JN: jet nebulizer. * *P* = 0.001, JN-L versus VMN-L; *P* = 0.017 JN-H versus VMN-L; ^†^ *P* < 0.001, JN-L and JN-H versus VMN-L; **^‡^** *P <* 0.001, JN-L and JN-H versus VMN-L; ^§^ *P* = 0.025, JN-L versus JN-H; ^‖^ *P* = 0.015, JN-H versus VMN-L.

**Table 2:** Particle size distribution of nebulized colistin distal to the endotracheal tube.

Variables	VMN-L	JN-L	JN-H	*P*-Value
MMAD (µm)	2.03 ± 0.24	2.09 ± 0.17	2.26 ± 0.05	0.434
GSD	1.58 ± 0.14	1.59 ± 0.10	1.58 ± 0.01	0.994
Fine particle % (1.1 to 4.7 µm)	87.83 ± 3.08	90.69 ± 4.55	95.83 ± 0.36	0.128

Note: Data are presented as means ± standard deviation; VMN: vibrating mesh nebulizer; JN: jet nebulizer; GSD: geometric standard deviation; MMAD: median aerodynamic diameter.

## References

[1] Wenzler E., Fraidenburg D.R., Scardina T., Danziger L.H. (2016). Inhaled Antibiotics for Gram-Negative Respiratory Infections. Clin. Microbiol. Rev..

[2] Boisson M., Jacobs M., Gregoire N., Gobin P., Marchand S., Couet W., Mimoz O. (2014). Comparison of intrapulmonary and systemic pharmacokinetics of colistin methanesulfonate (CMS) and colistin after aerosol delivery and intravenous administration of CMS in critically ill patients. Antimicro. Agents Chemother..

[3] Vardakas K.Z., Voulgaris G.L., Samonis G., Falagas M.E. (2018). Inhaled colistin monotherapy for respiratory tract infections in adults without cystic fibrosis: A systematic review and meta-analysis. Int. J. Antimicrob. Agents.

[4] Rodvold K.A., Yoo L., George J.M. (2011). Penetration of anti-infective agents into pulmonary epithelial lining fluid: Focus on antifungal, antitubercular and miscellaneous anti-infective agents. Clin. Pharmacokinet..

[5] Ehrmann S., Chastre J., Diot P., Lu Q. (2017). Nebulized antibiotics in mechanically ventilated patients: A challenge for translational research from technology to clinical care. Ann. Intensive Care.

[6] Sole-Lleonart C., Rouby J.J., Blot S., Poulakou G., Chastre J., Palmer L.B., Bassetti M., Luyt C.E., Pereira J.M., Riera J. (2017). Nebulization of Antiinfective Agents in Invasively Mechanically Ventilated Adults: A Systematic Review and Meta-analysis. Anesthesiology.

[7] Kofteridis D.P., Alexopoulou C., Valachis A., Maraki S., Dimopoulou D., Georgopoulos D., Samonis G. (2010). Aerosolized plus intravenous colistin versus intravenous colistin alone for the treatment of ventilator-associated pneumonia: A matched case-control study. Clin. Infect. Dis..

[8] Spapen H., Jacobs R., Van Gorp V., Troubleyn J., Honore P.M. (2011). Renal and neurological side effects of colistin in critically ill patients. Ann. Intensive Care.

[9] Lee Y.J., Wi Y.M., Kwon Y.J., Kim S.R., Chang S.H., Cho S. (2015). Association between colistin dose and development of nephrotoxicity. Crit. Care Med..

[10] Haworth C.S., Foweraker J.E., Wilkinson P., Kenyon R.F., Bilton D. (2014). Inhaled colistin in patients with bronchiectasis and chronic Pseudomonas aeruginosa infection. Am. J. Respir. Crit. Care Med..

[11] Guerin C., Fassier T., Bayle F., Lemasson S., Richard J.C. (2008). Inhaled bronchodilator administration during mechanical ventilation: How to optimize it, and for which clinical benefit?. J. Aerosol. Med. Pulm. Drug Deliv..

[12] Rello J., Rouby J.J., Sole-Lleonart C., Chastre J., Blot S., Luyt C.E., Riera J., Vos M.C., Monsel A., Dhanani J. (2017). Key considerations on nebulization of antimicrobial agents to mechanically ventilated patients. Clin. Microbiol. Infect..

[13] Boe J., Dennis J.H., Driscoll B.R., Bauer T.T., Carone M., Dautzenberg B., Diot P., Heslop K., Lannefors L. (2001). European Respiratory Society Task Force on the use of nebulizers. European Respiratory Society Guidelines on the use of nebulizers. Eur. Respir. J..

[14] Mc Callion O.N.M., Patel M.J. (1996). Viscosity effects on nebulisation of aqueous solutions. Int. J. Pharm..

[15] Finlay W.H., Lange C.F., King M., Speert D.P. (2000). Lung delivery of aerosolized dextran. Am. J. Respir. Crit. Care Med..

[16] Lu Q., Luo R., Bodin L., Yang J., Zahr N., Aubry A., Golmard J.L., Rouby J.J. (2012). Efficacy of high-dose nebulized colistin in ventilator-associated pneumonia caused by multidrug-resistant Pseudomonas aeruginosa and Acinetobacter baumannii. Anesthesiology.

[17] Kim Y.K., Lee J.H., Lee H.K., Chung B.C., Yu S.J., Lee H.Y., Park J.H., Kim S., Kim H.K., Kiem S. (2017). Efficacy of nebulized colistin-based therapy without concurrent intravenous colistin for ventilator-associated pneumonia caused by carbapenem-resistant Acinetobacter baumannii. J. Thorac. Dis..

[18] Athanassa Z.E., Markantonis S.L., Fousteri M.Z., Myrianthefs P.M., Boutzouka E.G., Tsakris A., Baltopoulos G.J. (2012). Pharmacokinetics of inhaled colistimethate sodium (CMS) in mechanically ventilated critically ill patients. Intensive Care Med..

[19] Hess D., Fisher D., Williams P., Pooler S., Kacmarek R.M. (1996). Medication nebulizer performance. Effects of diluent volume, nebulizer flow, and nebulizer brand. Chest.

[20] Li J., Milne R.W., Nation R.L., Turnidge J.D., Coulthard K. (2003). Stability of colistin and colistin methanesulfonate in aqueous media and plasma as determined by high-performance liquid chromatography. Antimicrob. Agents Chemother..

[21] Wallace S.J., Li J., Rayner C.R., Coulthard K., Nation R.L. (2008). Stability of colistin methanesulfonate in pharmaceutical products and solutions for administration to patients. Antimicrob. Agents Chemother..

[22] Ari A., Atalay O.T., Harwood R., Sheard M.M., Aljamhan E.A., Fink J.B. (2010). Influence of nebulizer type, position, and bias flow on aerosol drug delivery in simulated pediatric and adult lung models during mechanical ventilation. Respir. Care.

[23] Ge H.Q., Wang J.M., Lin H.L., Fink J.B., Luo R., Xu P., Ying K. (2019). Effect of Nebulizer Location and Spontaneous Breathing on Aerosol Delivery During Airway Pressure Release Ventilation in Bench Testing. J. Aaerosol. Med. Pulm. Drug Deliv..

[24] Wan G.H., Lin H.L., Fink J.B., Chen Y.H., Wang W.J., Chiu Y.C., Kao Y.Y., Liu C. (2014). In vitro evaluation of aerosol delivery by different nebulization modes in pediatric and adult mechanical ventilators. Respir. Care.

[25] Zhang G., David A., Wiedmann T.S. (2007). Performance of the vibrating membrane aerosol generation device: Aeroneb Micropump Nebulizer. J. Aerosol. Med. Pulm. Drug Deliv..

[26] Ghazanfari T., Elhissi A.M., Ding Z., Taylor K.M. (2007). The influence of fluid physicochemical properties on vibrating-mesh nebulization. Int. J. Pharm..

[27] Birkun A. (2014). Exogenous pulmonary surfactant as a vehicle for antimicrobials: Assessment of surfactant-antibacterial interactions in vitro. Scientifica.

[28] Ratjen F., Rietschel E., Kasel D., Schwiertz R., Starke K., Beier H., van Koningsbruggen S., Grasemann H. (2006). Pharmacokinetics of inhaled colistin in patients with cystic fibrosis. J. Antimicrob. Chemother..

[29] Katz S.L., Ho S.L., Coates A.L. (2001). Nebulizer choice for inhaled colistin treatment in cystic fibrosis. Chest.

[30] Miller D.D., Amin M.M., Palmer L.B., Shah A.R., Smaldone G.C. (2003). Aerosol delivery and modern mechanical ventilation: In vitro/in vivo evaluation. Am. J. Respir. Crit. Care Med..

[31] Yang S.H., Yang T.M., Lin H.L., Tsai Y.H., Fang T.P., Wan G.H. (2018). Size distribution of salbutamol/ipratropium aerosols produced by different nebulizers in the absence and presence of heat and humidification. Pulm. Pharmacol. Ther..

[32] Haddrell A.E., Davies J.F., Miles R.E., Reid J.P., Dailey L.A., Murnane D. (2014). Dynamics of aerosol size during inhalation: Hygroscopic growth of commercial nebulizer formulations. Int. J. Pharm..

[33] Lee A.K., Ling T.Y., Chan C.K. (2008). Understanding hygroscopic growth and phase transformation of aerosols using single particle Raman spectroscopy in an electrodynamic balance. Faraday Discuss.

[34] Steckel H., Eskandar F. (2003). Factors affecting aerosol performance during nebulization with jet and ultrasonic nebulizers. Eur. J. Pharm. Sci..

[35] Najlah M., Vali A., Taylor M., Arafat B.T., Ahmed W., Phoenix D.A., Taylor K.M., Elhissi A. (2013). A study of the effects of sodium halides on the performance of air-jet and vibrating-mesh nebulizers. Int. J. Pharm..

